# Green tea improves cognitive function through reducing AD-pathology and improving anti-oxidative stress capacity in Chinese middle-aged and elderly people

**DOI:** 10.3389/fnagi.2022.919766

**Published:** 2022-08-05

**Authors:** Ran Zhang, Lei Zhang, Zeng Li, Ping Zhang, Hao Song, Dong-ai Yao, Jing Cao, Jun-jian Zhang

**Affiliations:** Department of Neurology, Zhongnan Hospital of Wuhan University, Wuhan, China

**Keywords:** green tea, cognitive function, AD pathology, β-amyloid, tau, oxidative stress

## Abstract

**Background:** Numerous benefits of green tea have been reported. However, the effects of green tea on cognitive function remain disputable and the mechanism is still unclear.

**Objective:** To investigate the relationship of green tea consumption with cognitive function and related blood biomarkers among Chinese middle-aged and elderly people.

**Methods:** A total of 264 participants aged 50–70 years old were enrolled from Zhongnan Hospital of Wuhan University. They were interviewed about green tea consumption patterns and underwent neuropsychological tests covering five main cognitive domains to assess cognition including Montreal Cognitive Assessment (MoCA) and the other 10 scales. Then we detected serum oxidative stress biomarkers including Superoxide Dismutase (SOD), Malondialdehyde (MDA), Glutathione Peroxidase (GPx), Glutathione Reductase (GR), and Alzheimer’s disease (AD) markers including β-amyloid (Aβ)_40_, Aβ_42_, and phosphorylated tau-181 (pTau_181_).

**Results:** In the tea-consuming group, the MoCA scores (*P* = 0.000), Hopkins Verbal Learning Test (HVLT) immediate recall (*P* = 0.012) and delayed recall (*P* = 0.013) were significantly higher while Trail Making Test-B (*P* = 0.005) and Victoria Stroop test interference (*P* = 0.000) were lower. In terms of oxidative stress markers, the tea-consuming group had lower serum MDA levels (*P* = 0.002) and higher serum SOD (*P* = 0.005) and GPx (*P* = 0.007) levels. In terms of AD markers, serum pTau_181_ (P < 0.000), Aβ_42_ (*P* = 0.019) and total Aβ levels (*P* = 0.034) but not serum Aβ_40_ levels, were lower in the tea-consuming group. In the logistic regression analysis, there was a significant negative correlation between green tea consumption and cognitive impairment (OR = 0.26, 95 % CI 0.13 0.52 for high group).

**Conclusion:** Regular green tea consumption is associated with better cognitive function among Chinese middle-aged and elderly people, mainly reflected in memory and executive function. It may achieve protective effects by reducing AD-related pathology and improving anti-oxidative stress capacity and higher levels of tea consumption have a stronger protective effect.

## Introduction

Mild cognitive impairment (MCI) is an intermediate state between normal aging and dementia and is characterized by a mild decline of cognitive function while retaining intact functioning in daily life (Vega and Newhouse, 2014). As a high-risk factor for subsequent development of dementia, it should be paid more attention to its identification and early intervention. Previous studies have demonstrated that certain dietary patterns may have cognitive benefits, such as the Dietary Approaches to Stop Hypertension (van den Brink et al., 2019), Mediterranean diet (Petersson and Philippou, 2016), and so on. Among them, the consumption of green tea is very common in dietary patterns. Chinese people especially enjoy green tea. Numerous benefits of green tea have been reported (Ishii et al., 2019), including anti-cancer, cardiovascular protective, anti-inflammatory, anti-diabetic effects, etc.

However, the effects of green tea consumption on cognitive function remain disputable. The reasons may be as follows. First of all, the evaluation method of cognitive function is relatively single and there is no further analysis of the specific cognitive domains affected by green tea consumption. The Mini-Mental State Examination scale, used in most studies, is less sensitive in the early screening of cognitive impairment. And the influence of anxiety and depression has not been ruled out in many pieces of research. Furthermore, most studies in this area are foreign subject groups. The duration of tea consumption intervention is only a few months to years. Observational studies also rarely take the factor of consumption years into account. While different from foreign lifestyles and cultures, most Chinese people have the habit of green tea consumption for a long time and the consumption population in China is much larger than that in other countries. But there are very few studies on the Chinese cohorts (Noguchi-Shinohara et al., 2014; Schmidt et al., 2014; Ide et al., 2016; Tomata et al., 2016; Fischer et al., 2018; Liu et al., 2018). Therefore, the main purpose of this study is to analyze the relationship between green tea consumption and cognitive function among middle-aged and elderly people in China.

At the same time, we also hope to explore its possible mechanism. As a well-known antioxidant, polyphenols are the main biologically active component of green tea, in which the most abundant and active constituent is epigallocatechin-3-gallate (EGCG; Farzaei et al., 2019). Previous animal experiments have shown that green tea may improve cognitive function through various paths. The endogenous antioxidant defense mechanism (Burckhardt et al., 2008; Xu et al., 2010; Baluchnejadmojarad and Roghani, 2011; Jelenkovic et al., 2014; Schimidt et al., 2017) and anti-pathology effects of Alzheimer’s Disease (AD) was considered the most important (Rezai-Zadeh et al., 2008; Lee et al., 2009; Smith et al., 2010; Lopez del Amo et al., 2012; Wei et al., 2019; Ma et al., 2020; Sonawane et al., 2020).

In terms of oxidative stress, superoxide dismutase (SOD) acts as one of the key enzymatic antioxidant defenses against superoxide radicals. Malondialdehyde (MDA) is the principal end-product in the lipid peroxidation process and is cytotoxic (Xu et al., 2010). Glutathione peroxidase (GPx) is an important peroxide decomposing enzyme widely present in the body (Biasibetti et al., 2013). Glutathione reductase (GR) functions in catalyzing an oxidized form of glutathione into a reduced form. They play an essential role in preventing the oxidative decomposition of hemoglobin, maintaining the activity of sulfhydryl proteins, ensuring the reducibility of sulfhydryl proteins, and cell integrity (Rubio-Perez et al., 2016). Therefore, our study selected the four biomarkers above for testing.

Two main hypotheses were suggested for the primary AD pathogenesis (Torres et al., 2021; Trejo-Lopez et al., 2021). One is the amyloid cascade hypothesis. Amyloid-β (Aβ) deposition is widely accepted as central to AD pathology, of which Aβ_40_ and Aβ_42_ are the most common subtypes in the human body. The other hypothesis refers to abnormal phosphorylation of tau. Tau is a microtubule-associated protein, while hyperphosphorylation of tau leads to the formation of neurofibrillary tangles. In recent years, phosphorylated tau-181 (pTau_181_) is considered to be a potential AD blood marker, which may predict the longitudinal progress of AD (Janelidze et al., 2020). Accordingly, the three indicators above, namely Aβ_40_, Aβ_42_, and pTau_181_, were selected for detection in terms of anti-AD pathology.

We aim to examine the subjects’ serum oxidative stress and AD biomarkers and further analyze the relationship between green tea consumption and related blood markers, thereby exploring its possible mechanism.

## Materials and Methods

### Study population

A total of 386 subjects were screened at the Department of Neurology and Physical Examination Center in Zhongnan Hospital of Wuhan University from October 2019 to December 2020. The inclusion criteria included subjects: (1) aged 50–70 years old, (2) who had received at least primary education, and (3) who could provide voluntary written informed consent. Subjects were excluded who: (1) had a recent record of severe cerebrovascular disease found on MRI or CT, (2) have been diagnosed with dementia according to DSM-V (American-Psychiatric-Association, 2013), (3) showed obvious auditory or visual handicaps, or (4) suffered from other severe diseases that significantly affect cognitive function including the depression or anxiety status, thyroid disease, infection, tumor, or systemic disease.

**Figure 1:**
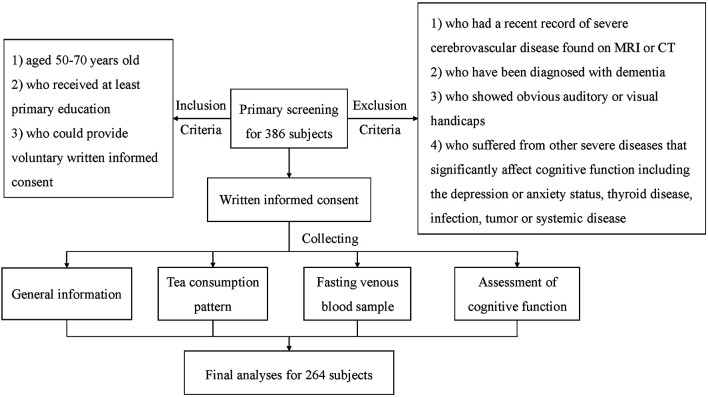
Participant flowchart. MCI, Mild cognitive impairment.

In this study, participants were interviewed by one specific staff to acquire data regarding demographic information, medical history, and green tea consumption. Thereafter they received an assessment of cognitive function at the memory outpatient clinic. Finally, 5 ml of fasting venous blood sample was collected from each subject. After excluding subjects who did not complete the full set of cognitive assessments (68 subjects) or lacked blood samples (106 subjects), 264 subjects were admitted to the final analyses, including 138 males and 126 females, with an average age of 60.8 years. This research has obtained all participants’ written informed consent and ethics approval (ClinicalTrials.gov ID: NCT04999813). See participant flowchart (Figure 1).

### Demographic information, tea consumption patterns, and medical history

Tea consumption information was obtained by direct interviewing with the following four questions: (Q1) the type of tea most commonly consumed (green tea, black tea, oolong tea, or others), (Q2) the consumption frequency (days per week), (Q3) the daily volume of tea (cups per day), and (Q4) the duration of tea consumption habit (years). A cup of standard green tea has a volume of 100 ml.

First, we divided the subjects into two groups for preliminary analysis. The tea-consuming group was defined as having at least 1 cup/day for 1 year or more and others are classified as the non-consumption group. Furthermore, the tea-consuming group was divided based on the consumption frequency and daily volume of tea: 1–2 cups/day (low), 3–9 cups/day (medium), and ≥10 cups/day (high; Shen et al., 2015). Accordingly, all subjects fell into four groups, namely “none, low, medium, and high”, for subgroup analysis.

The following demographic information was collected including age, gender, education level (elementary school/junior high school/high school/university and above), weight, height, smoking history (no or yes), alcohol consumption (no or yes), and physical activity scores (Fischer et al., 2018). Body mass index (BMI) was calculated as weight (kg) divided by the square of height (m). Participants also reported if they had a history of hypertension, hypercholesterolemia, diabetes, or atrial fibrillation. All the information above was determined by the same researcher. Fasting blood glucose, total cholesterol, triglyceride, and homocysteine levels were measured by the laboratory department of Zhongnan hospital. These characteristics were identified because they are risk factors for cognitive function or have effects on cognitive function and can be potential confounders.

### Assessment of cognitive function

Every subject underwent a battery of neuropsychological tests to assess cognitive functions covering the five main cognitive domains of memory, language, attention, executive function, and visual space (Table 1 for details), all completed by the same experienced memory clinicians.

**Table 1:** The battery of neuropsychological tests.

**Cognitive domain**	**Scale**
Global function	Montreal Cognitive Assessment (MoCA)
Memory	Hopkins Verbal Learning Test (HVLT)
Language	Verbal Fluency Test
Attention	Symbol Digit Modalities Test, Trail Making Test-A (TMT-A)
Executive function	Victoria Stroop Test (VST), Trail Making Test-B (TMT-B)
Visual space	Clock Drawing Test
Affective disorders	Hamilton Anxiety Rating Scale, Hamilton Depression Rating Scale
Functional autonomy	Instrumental Activities of Daily Living (IADL)

The Montreal Cognitive Assessment (MoCA) is a screening test assessing the global cognitive function that covers memory, visuospatial ability, executive function, attention, concentration, working memory, and orientation. At present, there are still different reports on the normal value of MoCA. Our study used ≥24 points as normal based on the community-based large sample survey conducted by Jia Jian-ping (Guo and Hong, 2012).

The Hopkins Verbal Learning Test-Revised (HVLT-R) consists of immediate recall after three consecutive learning, delayed recall, and delayed recognition after 20-min intervals used to measure verbal memory in all participants. The Verbal Fluency Test requires the subject to list as many examples as possible in 1 min in a certain category. The three categories in our study used to evaluate language function are animals, fruits, and vegetables. Trail Making Test (TMT) is divided into two parts, A and B. Part A requires the subjects to connect the circled numbers from 1 to 25 in sequence. In Part B, the circles include both numbers (one-13) and Chinese characters (One-12) and the subject should draw a line to connect alternative numbers and characters in ascending order (i.e., 1-One-2-Two-3-Three). TMT-A and B are aimed to assess attention and executive function. The Symbol Digit Modalities Test contains a series of numbers between one and nine. Subjects are required to fill in the corresponding symbol based on a digit-symbol key provided within 90 s to measure their attention and processing speed. Victoria Stroop Test (VST) consists of three trials: a word, a color, and a word-color interference trial. Subjects need to read the colors in each card as quickly as possible. Interference scores were calculated as the time difference for completion of the interference measures minus the non-interference tasks, used to assess executive function. Clock Drawing Test requires subjects to draw a rounded clock on white article, fill in all the numbers, and indicate a certain time 11:10 to measure visual space ability. Hamilton Anxiety Rating Scale and Hamilton Depression Rating Scale were used to exclude subjects with moderate to severe anxiety or depression. Instrumental Activities of Daily Living is mainly used to assess the subjects’ ability of daily living, especially focusing on the ability to use tools in life, thereby estimating the severity of cognitive impairment. Thus, subjects who have developed dementia are excluded.

### Measurements of serum biomarkers levels

For each participant, a fasting blood sample was collected from 6:00 to 7:00 in the morning and stored at −80°C after centrifugation and sub packaging. AD markers were all measured with enzyme-linked immunosorbent assay (ELISA). Serum Aβ_40_ and Aβ_42_ levels were detected by human Aβ_40_ and Aβ_42_ ELISA kits (Reddot, British Columbia, Canada), and serum pTau_181_ levels were determined by human phosphorylated Tau_181_ Protein ELISA kit (Jianglai Biological, Shanghai, China). Oxidative stress markers were all measured with biochemical kits (Nanjing Jiancheng, Nanjing, China). The whole detection was used in a blinded manner with respect to subject information.

### Statistical analysis

Gender, education level, smoking history, alcohol consumption, history of hypertension, hypercholesterolemia, diabetes, and atrial fibrillation were considered to be categorical variables. These were shown as the number of cases (percentage) and analyzed by the chi-square test. The remaining items were continuous variables and presented as mean ± standard deviation (Mean ± SD). Two independent-sample t-tests werea used for comparisons between two groups of normal distribution and one-way analysis of variance was used among multiple groups. Finally, we adopted logistic regression analysis to evaluate the correlation between green tea consumption and MCI, following adjustments for individual characteristics. *P* <0.05 was defined as being statistically significant and statistical testing was two-tailed. Statistical analyses were conducted using SPSS 17.0 software (SPSS Inc., Chicago, IL, USA).

## Results

### Characteristics of the study population

**Figure 2:**
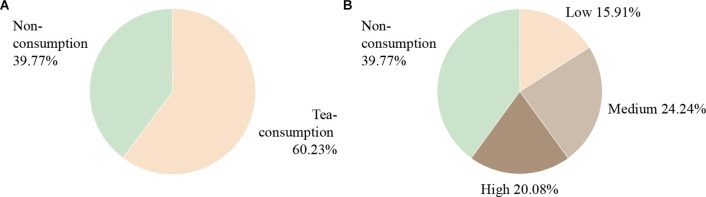
Participants’ grouping profile. **(A)** First, the 264 subjects were divided into two groups for preliminary analysis, including 105 cases (39.77%) for the non-consumption group and 159 cases (60.23%) for the tea-consuming group. **(B)** Furthermore, the tea-consuming group was divided into “low, medium, and high” groups based on the consumption frequency and daily volume of tea for subgroup analysis. There were 42 cases (15.91 %) in the low group, 64 cases (24.24%) in the medium group, and 53 cases (20.08%) in the high group.

The characteristics of the 264 participants are shown in Table 2, Figure 2A with 105 cases (39.77%) for the non-consumption group and 159 cases (60.23%) for the tea-consuming group. Differences were observed in gender (41.9% of males in the non-consumption group vs. 59.1% in the tea-consuming group, *P* = 0.006), smoking history (11.4% vs. 22.6%, *P* = 0.021), and physical activity scores (5.2 ± 2.0 vs. 4.7 ± 1.9, *P* = 0.034) between the two groups. There were more males, more smokers, and lower physical activity scores in the tea-consuming group, which is consistent with the social phenomenon that middle-aged and elderly men prefer tea consumption. And the trend of smoking may be related to gender. There was no significant difference in other demographic characteristics and hematological findings (*P* > 0.05).

**Table 2:** Demographic and clinical characteristics of tea-consuming and non-consuming group.

	**Non-consumption group (*n* = 105)**	**Tea-consuming group (*n* = 159)**	***P* value**
Age, years (SD)	61.5 (5.5)	60.3 (5.6)	0.075
Male (%)	44 (41.9)	94 (59.1)	0.006*
Education			0.669
≥High school (%)	93 (88.6)	138 (86.8)	
<High school (%)	12 (11.4)	21 (13.2)	
BMI (SD)	23.31 (2.77)	23.22 (2.84)	0.806
Systolic blood pressure (SD)	131.6 (15.6)	132.4 (15.5)	0.688
Diastolic blood pressure (SD)	77.9 (11.0)	78.1 (10.6)	0.887
Hypertension (%)	36 (34.3)	53 (33.3)	0.873
Diabetes mellitus (%)	14 (13.3)	22 (21.7)	0.907
Hypercholesterolemia (%)	25 (23.8)	38 (23.9)	0.987
Atrial fibrillation (%)	4 (3.8)	16 (10.1)	0.060
Smoking history (%)	12 (11.4)	36 (22.6)	0.021*
Alcohol consumption (%)	18 (17.1)	20 (12.6)	0.301
Physical activity score (SD)	5.2 (2.0)	4.7 (1.9)	0.034*
Fasting Glucose, mmol/L (SD)	5.93 (1.16)	5.84 (1.60)	0.633
Cholesterol, mmol/L (SD)	4.91 (1.05)	5.05 (1.07)	0.303
Triglyceride, mmol/L (SD)	1.52 (0.32)	1.61 (0.39)	0.056
Homocysteine, umol/L (SD)	13.91 (2.53)	13.32 (2.56)	0.090
Mild Cognitive Impairment (%)	66 (62.9)	72 (45.3)	0.005*

In the non-consumption group, there were 66 MCI patients and 39 cognitively normal subjects. In the tea consumption group, there were 72 MCI patients and 87 cognitively normal subjects. SD, standard deviation; BMI, Body mass index. All continuous variables are presented as the mean (SD). Two independent-sample t-tests and chi-square test were used for comparisons between the two groups. *P < 0.05 was defined as being statistically significant.

In subgroup analysis, there were 105 cases (39.77%) in the non-consumption group, 42 cases (15.91%) in the low group, 64 cases (24.24%) in medium, and 53 cases (20.08%) in high, as shown in Figure 2B. The results were similar to the above in that differences in gender (*P* = 0.046), smoking history (*P* = 0.027), and physical activity scores (*P* = 0.020) were observed, as presented in Table 3 for details. Compared with the non-consumption group, the medium and high groups had more males and smokers, but no more for physical activity scores.

**Table 3:** Demographic and clinical characteristics of subgroup analysis.

	**None (*n* = 105)**	**Low (*n* = 42)**	**Medium (*n* = 64)**	**High (*n* = 53)**	***P* value**
Age, years (SD)	61.5 (5.5)	59.9 (4.9)	60.3 (5.5)	60.6 (6.2)	0.315
Male (%)	44 (41.9)	22 (52.4)	40 (62.5)	31 (58.5)	0.046*
Education					0.790
≥High school (%)	93 (88.6)	38 (90.5)	54 (84.4)	46 (86.8)	
<High school (%)	12 (11.4)	4 (9.5)	10 (15.6)	7 (13.2)	
BMI (SD)	23.31 (2.77)	22.97 (2.79)	23.01 (2.75)	23.67 (2.98)	0.549
Systolic blood pressure (SD)	131.6 (15.6)	130.9 (13.1)	134.5 (17.7)	131.1 (14.5)	0.558
Diastolic blood pressure (SD)	77.9 (11.0)	79.3 (10.3)	76.7 (11.3)	78 (9.8)	0.611
Hypertension (%)	36 (34.3)	16 (38.1)	21 (32.8)	16 (30.2)	0.875
Diabetes mellitus (%)	14 (13.3)	5 (11.9)	13 (20.3)	6 (11.3)	0.469
Hypercholesterolemia (%)	25 (23.8)	6 (14.3)	19 (29.7)	13 (24.5)	0.344
Atrial fibrillation ( %)	4 (3.8)	2 (4.8)	7 (10.9)	4 (7.5)	0.301
Smoking history (%)	12 (11.4)	6 (14.3)	18 (28.1)	13 (24.5)	0.027*
Alcohol consumption (%)	18 (17.1)	5 (11.9)	10 (15.6)	8 (15.1)	0.888
Physical activity score (SD)	5.2 (2.0)	4.4 (1.6)	5.1 (2.0)	4.4 (1.9)	0.020*
Fasting Glucose, mmol/L (SD)	5.93 (1.16)	5.86 (1.20)	5.76 (1.57)	5.93 (1.90)	0.883
Cholesterol, mmol/L (SD)	4.91 (1.05)	5.10 (0.93)	5.06 (1.18)	5.01 (1.06)	0.751
Triglyceride, mmol/L (SD)	1.52 (0.32)	1.60 (0.36)	1.58 (0.43)	1.65 (0.38)	0.184
Homocysteine, umol/L (SD)	13.91 (2.53)	13.21 (2.49)	12.96 (2.56)	13.84 (2.59)	0.127

The tea-consuming group was divided into “low, medium, and high” groups based on the consumption frequency and daily volume of tea for subgroup analysis. One-way analysis of variance and chi-square test was used for comparisons among multiple groups. *P < 0.05 was defined as being statistically significant.

### The correlation between green tea consumption and cognition

**Figure 3:**
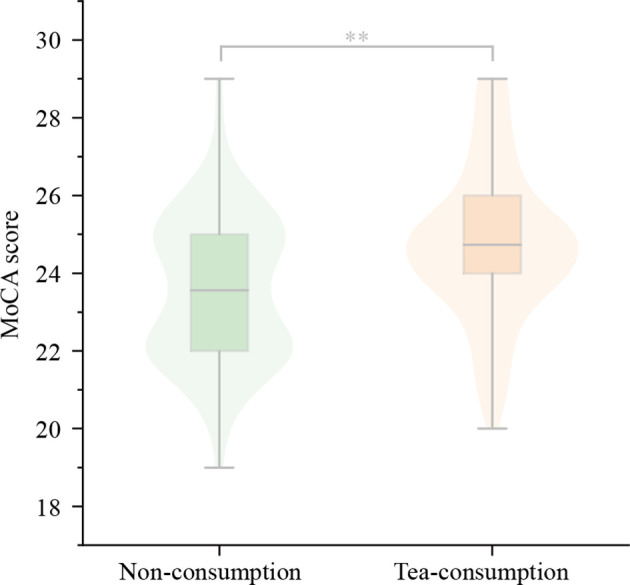
The difference for MoCA score between non-consuming group and tea-consuming group. The MoCA score was higher in the tea-consuming group significantly. MoCA, Montreal Cognitive Assessment scale. ^**^P < 0.001.

There were significant differences for MoCA (23.6 ± 1.9 vs. 24.7 ± 2.0, *P* = 0.000), HVLT immediate recall (19.2 ± 4.3 vs. 20.6 ± 4.7, *P* = 0.012), HVLT delayed recall (5.9 ± 2.1 vs. 6.6 ± 2.0, *P* = 0.013), HVLT delayed recognition (20.4 ± 2.3 vs. 21.0 ± 2.1, *P* = 0.040), TMT-B (83.79 ± 16.56 vs. 78.12 ± 15.68, *P* = 0.005), and VST interference (13.99 ± 1.78 vs. 13.16 ± 1.80, *P* = 0.000) between non-consumption group and tea-consuming group. The MoCA, HVLT immediate recall, delayed recall, and delayed recognition scores were higher while the TMT-B time and VST interference time were shorter in the tea-consuming group, as shown in Table 4; Figure 3. It is suggested that green tea consumption has a protective effect on cognitive function in a way, which is mainly reflected in the memory and executive function domain.

**Table 4:** The cognition differences between the non-consuming group and tea-consuming group.

		Non-consumption group (*n* = 105)	Tea-consuming group (*n* = 159)	*P* value
Global	MoCA (SD)	23.6 (1.9)	24.7 (2.0)	0.000*
Memory	HVLT immediate recall (SD)	19.2 (4.3)	20.6 (4.7)	0.012*
	HVLT delayed recall (SD)	5.9 (2.1)	6.6 (2.0)	0.013*
	HVLT delayed recognition (SD)	20.4 (2.3)	21.0 (2.1)	0.040*
Language	Verbal Fluency Test (SD)	45.6 (9.7)	44.5 (8.2)	0.304
Attention	SDMT (SD)	39.1 (11.2)	41.4 (10.5)	0.086
	TMT-A, s (SD)	46.12 (12.33)	43.22 (12.24)	0.062
Executive-function	TMT-B, s (SD)	83.79 (16.56)	78.12 (15.68)	0.005*
	VST interference, s (SD)	13.99 (1.78)	13.16 (1.80)	0.000*
Visual space	CDT (SD)	10.6 (1.8)	11.0 (1.9)	0.058
Affective-disorder	HAMA (SD)	2.2 (1.6)	2.5 (1.8)	0.197
	HAMD (SD)	3.4 (1.9)	3.4 (1.9)	0.950

MoCA, Montreal Cognitive Assessment; HVLT, Hopkins Verbal Learning Test; SDMT, Symbol Digit Modalities Test; TMT, Trail Making Test; VST, Victoria Stroop Test; CDT, Clock Drawing Test; HAMA, Hamilton Anxiety Rating Scale; HAMD, Hamilton Depression Rating Scale. *P < 0.05 was defined as being statistically significant.

**Figure 4:**
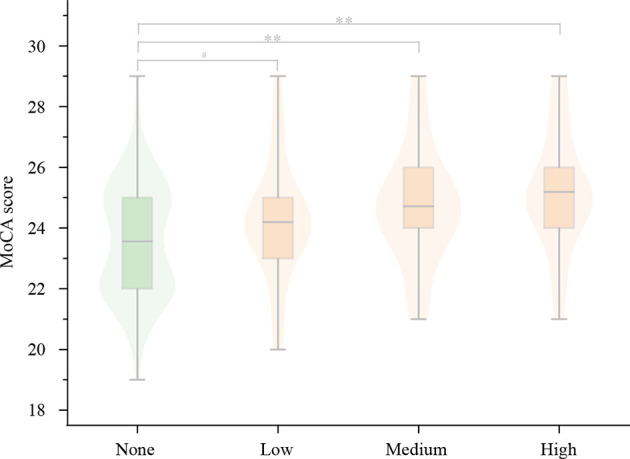
The results of subgroup analysis for MoCA scores. Compared with the non-consuming group, the MoCA score gradually increased in the low, medium, and high group, of which there was a significant difference only in the medium and high group, but not in the low group. MoCA, Montreal Cognitive Assessment scale. ^**^P < 0.001; ^#^*P* ≥ 0.05.

The results of subgroup analysis were similar to the above difference that was mainly reflected in memory and executive function. Compared with the non-consumption group, the low group generally had no significant difference. The MoCA scores (25.2 ± 1.9 vs. 23.6 ± 1.8, *P* = 0.000) and HVLT immediate recall (21.3 ± 4.7 vs. 19.2 ± 4.3, *P* = 0.033) were higher in the high group while the TMT-B time (76.26 ± 13.41 vs. 83.79 ± 16.56, *P* = 0.029) and VST interference time (12.98 ± 1.80 vs. 13.99 ± 1.78, *P* = 0.006) were shorter, as shown in Table 5, Figure 4. Probably, higher tea consumption frequency and volume have a stronger protective effect on cognitive function.

**Table 5:** The cognition differences of subgroup analysis.

	**None (*n* = 105)**	**Low (*n* = 42)**	**Medium (*n* = 64)**	**High (*n* = 53)**	***P* value**
MoCA (SD)	23.6 (1.8)	24.2 (1.9)	24.7 (1.9)	25.2 (1.9)	0.000*
HVLT immediate recall (SD)	19.2 (4.3)	19.9 (4.6)	20.6 (4.7)	21.3 (4.7)	0.038*
HVLT delayed recall (SD)	5.9 (2.1)	6.3 (2.1)	6.6 (2.1)	6.8 (2.0)	0.047*
HVLT delayed recognition (SD)	20.4 (2.3)	20.8 (2.1)	21.2 (1.9)	20.9 (2.3)	0.164
Verbal Fluency Test (SD)	45.6 (9.7)	43.9 (7.5)	46.2 (8.7)	42.9 (7.7)	0.144
SDMT (SD)	39.1 (11.2)	41.2 (10.3)	40.7 (10.7)	41.4 (10.6)	0.303
TMT-A, s (SD)	46.12 (12.33)	44.30 (12.43)	43.37 (12.48)	42.19 (11.93)	0.242
TMT-B, s (SD)	83.79 (16.56)	80.59 (18.39)	78.04 (15.54)	76.26 (13.41)	0.024*
VST interference, s (SD)	13.99 (1.78)	13.42 (1.90)	13.14 (1.73)	12.98 (1.80)	0.002*
CDT (SD)	10.6 (1.8)	11.1 (1.9)	10.9 (1.9)	11.1 (1.8)	0.225
HAMA (SD)	2.2 (1.6)	2.4 (1.9)	2.6 (1.8)	2.4 (1.8)	0.555
HAMD (SD)	3.4 (1.9)	3.4 (2.1)	3.3 (1.8)	3.6 (1.8)	0.882

MoCA, Montreal Cognitive Assessment; HVLT, Hopkins Verbal Learning Test; SDMT, Symbol Digit Modalities Test; TMT, Trail Making Test; VST, Victoria Stroop Test; CDT, Clock Drawing Test; HAMA, Hamilton Anxiety Rating Scale; HAMD, Hamilton Depression Rating Scale. *P < 0.05 was defined as being statistically significant.

### The correlation between green tea consumption and serum biomarkers

**Figure 5:**
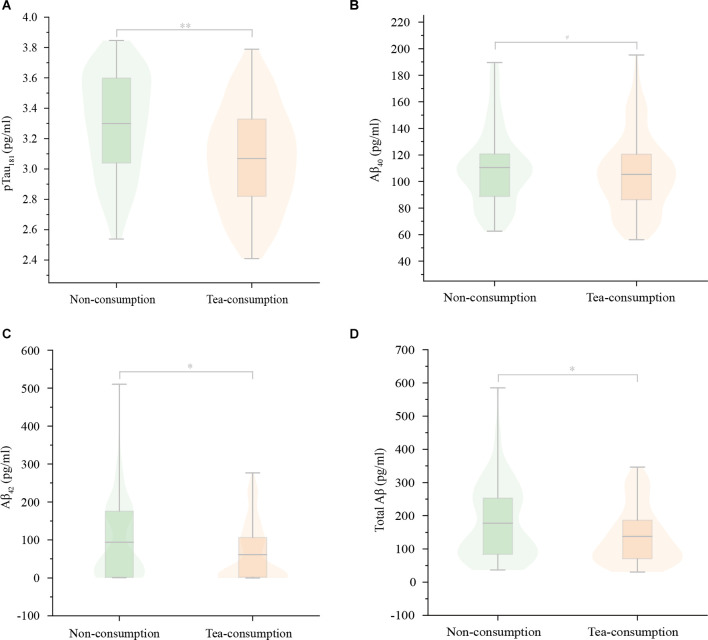
The differences in serum AD markers between the non-consuming group and the tea-consuming group. There were significant differences in serum pTau181 **(A)**, Aβ42 **(C)**, and total Aβ **(D)** levels between the two groups but no difference in serum Aβ40 **(B)** levels. The serum pTau181, Aβ42, and total Aβ levels were lower in the tea-consuming group. Aβ, Amyloid-β; pTau181, phosphorylated tau-181. ^**^P < 0.001; ^*^P < 0.05; ^#^*P* ≥ 0.05.

In terms of anti-oxidative stress, there were significant differences for serum SOD (44.73 ± 3.23 vs. 45.91 ± 3.31, *P* = 0.005), MDA (14.39 ± 1.53 vs. 13.79 ± 1.54, *P* = 0.002), and GPx levels (647.12 ± 45.72 vs. 661.99 ± 42.07, *P* = 0.007) between the non-consumption group and tea-consuming group, but no difference for serum GR levels. The tea-consuming group had lower serum MDA levels and higher serum SOD and GPx levels, as presented in Table 6, Figure 5.

**Table 6:** The biomarker differences between non-consuming group and tea-consuming group.

	**Non-consumption group (*n* = 105)**	**Tea-consuming group (*n* = 159)**	***P* value**
Serum pTau_181_, pg/ml (SD)	3.30 (0.33)	3.07 (0.33)	0.000*
Serum Aβ_40_, pg/ml (SD)	110.41 (28.88)	105.35 (28.71)	0.164
Serum Aβ_42_, pg/ml (SD)	82.19 (20.69)	75.87 (21.59)	0.019*
Serum total Aβ, pg/ml (SD)	192.59 (44.42)	181.21 (41.15)	0.034*
Serum Aβ_42/40_ ratio (SD)	0.77 (0.19)	0.75 (0.27)	0.798
Serum SOD, U/mL (SD)	44.73 (3.23)	45.91 (3.31)	0.005*
Serum MDA, nmol/mL (SD)	14.39 (1.53)	13.79 (1.54)	0.002*
Serum GR, U/L (SD)	40.06 (1.64)	40.49 (1.69)	0.068
Serum GPx, μmol/L (SD)	647.12 (45.72)	661.99 (42.07)	0.007*

pTau_181_, phosphorylated tau-181; Aβ, Amyloid-β; SOD, Superoxide dismutase; MDA, Malondialdehyde; GR, Glutathione reductase; GPx, Glutathione peroxidase. *P < 0.05 was defined as being statistically significant.

**Figure 6:**
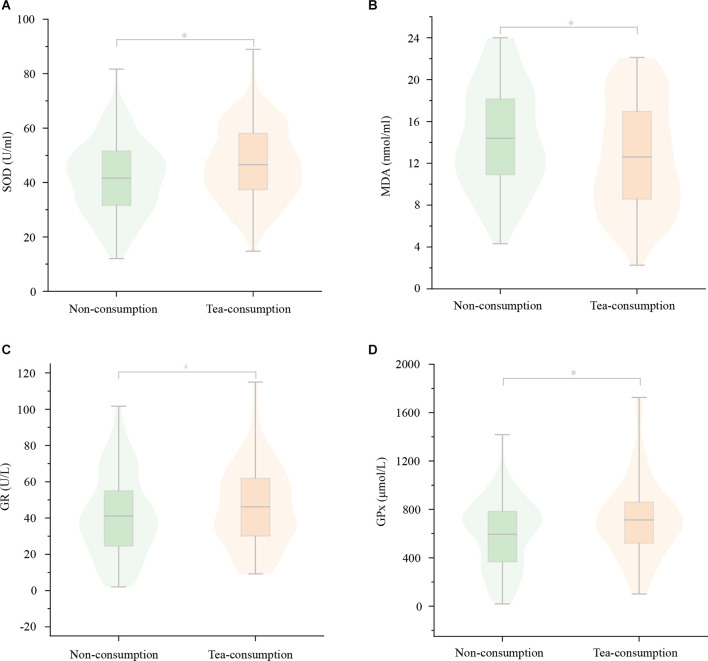
The differences in serum oxidative stress markers between the non-consuming group and tea-consuming group. There were significant differences in serum SOD **(A)**, MDA **(B)**, and GPx **(D)** levels between the two groups, but no difference in serum GR **(C)** levels. The tea-consuming group had lower serum MDA levels and higher serum SOD and GPx levels. SOD, superoxide dismutase; MDA, Malondialdehyde; GPx, Glutathione peroxidase; GR, Glutathione reductase. ^*^P < 0.05; ^#^*P* ≥ 0.05.

In terms of anti-AD pathology, there were significant differences for serum pTau_181_ (3.30 ± 0.33 vs. 3.07 ± 0.33, *P* < 0.000), Aβ_42_ (82.19 ± 20.69 vs. 75.87 ± 21.59, *P* = 0.019), and total Aβ levels (192.59 ± 44.42 vs. 181.21 ± 41.15, *P* = 0.034) between the two groups but no difference for serum Aβ_40_ levels and the ratio of Aβ_42/40_. The serum pTau_181_, Aβ_42_, and total Aβ levels in the tea-consuming group were lower, as presented in Table 6 and Figure 6. It can be seen that green tea consumption may protect cognitive function by reducing AD pathology and improving anti-oxidative stress capacity.

The trend of blood marker levels in subgroup analysis was similar to the above but slightly different. In terms of anti-oxidative stress, there were still differences for serum SOD (*P* = 0.023), MDA (*P* = 0.011), and GPx levels (*P* = 0.037) among the four groups. However, in the pairwise comparison for serum GR with the non-consumption group, the high group showed a significant difference (40.79 ± 1.94 vs. 40.06 ± 1.64, *P* = 0.045). The difference in the pairwise comparison was mainly reflected in the medium and high groups, where the serum MDA levels were lower while the SOD, GPx, and GR levels were higher, but no difference in the low group generally.

In terms of anti-AD pathology, there were still differences for serum pTau_181_ (*P* < 0.000), Aβ_42_ (*P* = 0.041), and total Aβ (*P* = 0.005) among the four groups. The difference in the pairwise comparison was also reflected in the medium and high group, where the serum pTau_181_, Aβ_42_, and total Aβ levels were lower. The results were displayed in Table 7 for details. Thus, high consumption of green tea is more likely to affect AD pathology and anti-oxidative stress capacity.

**Table 7:** The biomarker differences of subgroup analysis.

	**None (*n* = 105)**	**Low (*n* = 42)**	**Medium (*n* = 64)**	**High (*n* = 53)**	***P* value**
Serum pTau_181_, pg/ml (SD)	3.30 (0.33)	3.17 (0.33)	3.10 (0.33)	2.95 (0.31)	0.000*
Serum Aβ_40_, pg/ml (SD)	110.41 (28.88)	107.62 (26.54)	109.60 (32.57)	98.42 (24.23)	0.082
Serum Aβ_42_, pg/ml (SD)	82.19 (20.69)	79.05 (21.68)	76.11 (22.62)	73.06 (20.24)	0.041*
Serum total Aβ, pg/ml (SD)	192.59 (44.42)	186.67 (41.98)	185.70 (47.83)	171.47 (28.97)	0.005*
Serum Aβ_42/40_ ratio (SD)	0.77 (0.19)	0.75 (0.20)	0.73 (0.25)	0.80 (0.32)	0.645
Serum SOD, U/mL (SD)	44.73 (3.23)	45.40 (3.31)	45.95 (3.34)	46.26 (3.28)	0.023*
Serum MDA, nmol/mL (SD)	14.39 (1.53)	14.01 (1.72)	13.84 (1.31)	13.57 (1.64)	0.011*
Serum GR, U/L (SD)	40.06 (1.64)	40.42 (1.72)	40.30 (1.40)	40.79 (1.94)	0.073
Serum GPx, μmol/L (SD)	647.12 (45.72)	656.95 (41.78)	661.20 (42.74)	666.94 (41.74)	0.037*

pTau_181_, phosphorylated tau-181; Aβ, Amyloid-β; SOD, Superoxide dismutase; MDA, Malondialdehyde; GR, Glutathione reductase; GPx, Glutathione peroxidase. *P < 0.05 was defined as being statistically significant.

### Logistic regression analysis between green tea consumption and cognitive impairment

**Figure 7:**
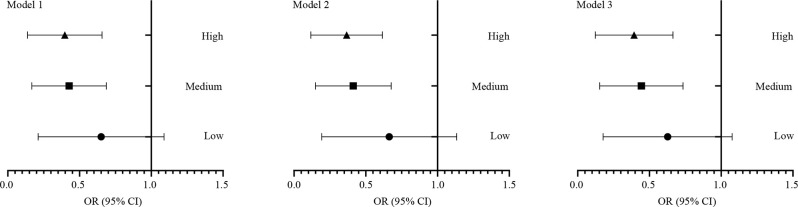
The logistic regression analysis between green tea consuming and cognitive impairment. All 264 subjects were divided again into the MCI group (MoCA < 24 scores) and the control group (MoCA ≥ 24 scores). Statistically, significant inverse associations were observed between green tea consuming and mild cognitive impairment. Model 1: Crude model. Model 2: Adjusted for age, gender, education, smoking history, alcohol consuming, BMI, and physical activity scores. Model 3: Adjusted for Model 2 + hypertension, diabetes, hyperlipidemia, and atrial fibrillation. OR, odds ratio; CI, confidence interval.

All 264 subjects were divided again into the MCI group (MoCA <24 scores) and the normal group (MoCA ≥ 24 scores). Statistically, significant inverse associations were observed between green tea consumption and mild cognitive impairment in logistic regression analysis, as shown in Table 8, Figure 7. With the non-consumption group as reference, the crude odds ratios of MCI for different groups were respectively 1.06 (95% CI 0.51 2.24) for the low group, 0.49 (95% CI 0.26 0.92) for medium, and 0.26 (95% CI 0.13 0.52) for high. We included a variety of potential confounders in our multivariate logistic models. However, the results did not change substantially even after adjustment for these variables.

**Table 8:** Odds ratios and 95% CIs from logistic regression models for the association between green tea consumption and mild cognitive impairment.

**Logistic regression models**	**None (*n* = 105)**	**Low (*n* = 42)**	***P* value**	**Medium (*n* = 64)**	***P* value**	**High (*n* = 53)**	***P* value**	**Total *P* value**
Model 1	1.00 (reference)	1.06 (0.51,2.24)	0.871	0.49 (0.26,0.92)	0.027	0.26 (0.13,0.52)	0.000	0.000
Model 2	1.00 (reference)	0.99 (0.46,2.16)	0.988	0.46 (0.24,0.90)	0.022	0.26 (0.12,0.53)	0.000	0.001
**Model 3**	1.00 (reference)	0.96 (0.44,2.12)	0.931	0.48 (0.24,0.94)	0.033	0.28 (0.13,0.59)	0.001	0.002

OR, odds ratio; CI, confidence interval. Model 1: Crude model. Model 2: Adjusted for age, gender, education, smoking history, alcohol consumption, BMI, and physical activity scores. Model 3: Adjusted for Model 2 + hypertension, diabetes, hyperlipidemia, and atrial fibrillation.

## Discussion

This study found that higher levels of green tea consumption may exert favorable effects on cognitive function and serum biomarkers of AD and oxidative stress in middle-aged and elderly adults.

As for the assessment of cognitive function, we used a battery of neuropsychological tests containing 11 scales and covering five prime cognitive domains. The results demonstrated that the MoCA scores of the tea-consuming group were significantly higher than that of the non-consumption group and were better as the frequency and volume of tea increased. In logistic regression analysis, there was a significant negative correlation between green tea consumption and MCI. The higher the levels of tea consumption, the lower the risk of cognitive impairment, which suggests that the protective effect of green tea on cognition may be related to frequency and volume.

Furthermore, green tea consumption mainly affected memory and executive function, which was also consistent with the neuropsychological evaluation of vascular cognitive impairment. The most common impaired cognitive domains in vascular cognitive impairment patients were processing speed and executive function (Van der Flier et al., 2018). It follows that green tea is likely to be an intervenable vascular protective factor by acting on cerebral blood vessels and its risk factors to achieve the effect of improving cognition. There have been many reports in the past on the vascular protective effects of green tea. Antioxidants, such as goji berries, green tea, thymus, and so on, may prevent cellular damage by reducing reactive oxygen species (ROS) overproduction or interfering in reactions that involve ROS. Increased production of ROS favors vascular dysfunction, inducing altered vascular permeability and inflammation, accompanied by the loss of vascular modulatory function, the imbalance between vasorelaxation and vasoconstriction, and the aberrant expression of inflammatory adhesion molecules (Bielli et al., 2015).

The mechanism underlying the association between green tea consumption and cognitive function may be concerned with endogenous antioxidant defense and anti-AD pathology. As mentioned above, previous evidence has indicated that, on the one hand, the intervention of green tea in animal models of cognitive impairment could change the levels of oxidative stress markers such as SOD, MDA, and glutathione and improve spatial learning and memory. On the other hand, green tea could inhibit the formation of Aβ plaques and Tau, thereby reversing the cognitive impairment of animals. In this study, we selected the seven classic markers both in the two aspects to be detected, adding new evidence to the mechanism demonstration from a clinical perspective.

In terms of anti-oxidative stress, our results were consistent with the past. The intervention of green tea reduced the cytotoxicity of MDA and increased the levels of SOD, GPx, and GR, which were pretty important enzymes to protect the structure and function of cell membranes from damage, indicating that green tea improves the ability to resist oxidative stress. Oxidative stress, which occurs when there is an imbalance between oxidant and antioxidant levels in the cell resulting in increased ROS production, is another important metabolic facet of AD pathology. Specifically, increased levels of ROS cause damage to macromolecules within the cell, and it is this damage of lipids, proteins, and nucleic acids that gives rise to pathological consequences. In the brain, ROS are eliminated by the free radical scavenger glutathione (GSH) through a chemical reaction that converts GSH to its oxidized state. As such, higher intracellular GSH levels protect cells from ROS-mediated insults. Given that neurons are particularly sensitive to oxidative damage due to the brain’s substantial metabolic requirements, alterations in GSH function can have profound effects on the brain and cognitive function (Song et al., 2021). Therefore, regular green tea consumption may improve cognitive function by increasing antioxidant capacity. And the higher the levels of consumption, the stronger the correlation for this mechanism.

In terms of anti-AD pathology, the serum Aβ_42_, total Aβ levels, and Aβ_42/40_ ratio in the tea-consuming group were lower but there was no difference in serum Aβ_40_ levels. A possible explanation is that though the content of Aβ_40_ is higher than Aβ_42_ in human cerebrospinal fluid and blood, Aβ_42_ has stronger toxicity and is easier to aggregate, thus forming the core of Aβ plaques and triggering neurotoxic effects. So, the sensitivity to drugs or interventions is higher than Aβ_40_. It can also be seen from existing research that the mechanism of green tea was more suited to Aβ_42_. For example, a molecular dynamics simulation experiment reported that EGCG could change the shape of Aβ_42_ through hydrogen bond interactions, damage its molecular dynamics and thereby disrupt Aβ_42_ protofibril (Zhan et al., 2020). Animal experiments have also revealed that EGCG significantly improved cognitive impairment in aged rats and reduced the formation of Aβ_42_ plaques in the brain (Wei et al., 2019). In addition, serum pTau_181_ demonstrated its potential as an early AD marker once again. It was first discovered that there was an association between serum pTau_181_ and green tea consumption, which also provides new ideas for our future research. In short, regular consumption of green tea may affect the formation of Aβ and Tau and thus protect cognition. And the higher the levels of consumption, the stronger the correlation for this mechanism.

In the meanwhile, it has been reported that oxidative stress plays a key role in the pathogenesis of AD. Aβ is responsible for the production of ROS, depletion of endogenous antioxidants, and increase in intracellular Ca^2+^ which further increases mitochondria dysfunctions, oxidative stress, release of pro-apoptotic factors, neuronal apoptosis, etc. Antioxidants are compounds that have the ability to counteract the oxidative damage conferred by ROS. Therefore, the antioxidant therapy may provide benefits and halt the progress of AD to advance stages by counteracting neuronal degeneration (Walia et al., 2022).

In conclusion, our study explored the relationship between green tea consumption and cognitive function among middle-aged and elderly people in China, as well as its anti-AD and oxidative stress effects. First, we comprehensively reflected on the green tea habits by inquiring about the frequency, the volume, and the years of consumption, and the subjects were divided into several groups accordingly. Next, the cognition assessment was more diversified. MoCA was used for overall screening that is more sensitive to MCI than the Mini-Mental State Examination scale. The battery of scales covered five prime domains including memory, language, attention, executive function, and visual space. And the influence of anxiety or depression was also excluded. Moreover, we converted the results of basic research into clinical evidence in the analysis of blood markers and the detection indicators were more comprehensive. Finally, maybe in contrast to Eastern and Western culture and diet patterns, most orientals are accustomed to long-term green tea consumption, in comparison to coffee in the West. Among the subjects of this study, the average consumption duration in the tea-consuming group was 23.3 ± 0.4 years. Consequently, the results of our study that green tea consumption was associated with better cognitive function may also be related to the years, adding new evidence to the argument for this issue. And specifically, for Chinese people’s eating habits, we recommend drinking more than 1,000 ml of green tea daily for more than 20 years.

The present study has some limitations. It was a cross-sectional study but determining the impact of diet on cognitive function is a long process. Our findings must be validated in a long-term follow-up study. Second, self-reporting is often compromised by amplifying “good” actions and minimizing “bad” ones and cannot avoid memory bias. Our researchers have tried their best to reduce the degree of bias in the process. Third, though there has been great progress and acceptance recently in the study of blood markers for AD, such as plasma Aβ_42/40_, neurofilament light chain protein, pTau_181_, and pTau_217_, their sensitivity and specificity are still much weaker than that in cerebrospinal fluid. If cerebrospinal fluid samples can be collected for analysis, stronger evidence will be obtained in the future. At last, the participants of our study were normal middle-aged and elderly people or those with MCI. Whether green tea consumption has a protective effect on patients with dementia is not yet known. These also provide us with future research directions.

## Conclusion

Green tea consumption is associated with better cognitive function, which is mainly reflected in memory and executive function. It might achieve protective effects by reducing AD-related pathology and improving anti-oxidative stress capacity. And higher levels of tea consumption may have a stronger protective effect on cognitive function.

## Data Availability Statement

The raw data supporting the conclusions of this article will be made available by the authors, without undue reservation.

## Ethics Statement

The studies involving human participants were reviewed and approved by ClinicalTrials.gov Protocol Registration and Results System (PRS) ID: NCT04999813. The patients/participants provided their written informed consent to participate in this study.

## Author Contributions

RZ: involved in study design, interviewing subjects, and drafting the manuscript. LZ: involved in study design, data analysis, and revising the manuscript. ZL: involved in collecting blood samples. PZ: involved in assessing cognitive function. HS: involved in detecting biomarkers. D-aY: involved in interviewing subjects. JC: involved in assessing cognitive function. J-jZ: involved in study design, revising the manuscript, and obtaining funding.

## Funding

This study was supported by National Natural Science Foundation of China (grant numbers 82071210 and 81771151, J-jZ).

## Abbreviations

MCI: Mild Cognitive Impairment
EGCG: Epigallocatechin-3-gallate
AD: Alzheimer’s Disease
SOD: Superoxide Dismutase
MDA: Malondialdehyde
GPx: Glutathione Peroxidase
GR: Glutathione Reductase
Aβ: β-amyloid
pTau_181_: phosphorylated Tau_181_
BMI: Body Mass Index
MoCA: Montreal Cognitive Assessment
HVLT: Hopkins Verbal Learning Test
TMT: Trail Making Test
VST: Victoria Stroop Test
ELISA: Enzyme Linked Immunosorbent Assay
ROS: Reactive Oxygen Species
GSH: Glutathione
SD: Standard Deviation
M: Mean
OR: Odds Ratio..
