# Supplementary material for: Green tea improves cognitive function through reducing AD-pathology and improving anti-oxidative stress capacity in Chinese middle-aged and elderly people
Source: Front Aging Neurosci. 2022 Aug 5;14:919766. doi: 10.3389/fnagi.2022.919766 (PMC9389233; doi:10.3389/fnagi.2022.919766)
Supplement: Supplementary file 1 [file Data_Sheet_1.PDF]

Supplementary Material

1 The cognition and biomarker differences between normal group and MCI group

All 264 subjects were further divided into the MCI group (MoCA<24 scores) and normal group (MoCA≥24 scores). There were 138 MCI patients and 126 cognitively normal subjects. Supplementary Table 1 showed the results of neuropsychological tests and biomarkers by normal and MCI group.

Supplementary Table 1 The cognition and biomarker differences between normal group and MCI group

		Normal group (n=126)	MCI group (n=138)	*P* value
Global	MoCA (SD)	25.9(1.3)	22.8(1.2)	0.000*
Memory	HVLT immediate recall (SD)	20.8(4.5)	19.3(4.6)	0.006*
	HVLT delayed recall (SD)	6.5(2.0)	6.1(2.1)	0.090
	HVLT delayed recognition (SD)	20.7(2.1)	20.8(2.3)	0.941
Language	Verbal Fluency Test (SD)	45.6(8.4)	44.3(9.1)	0.210
Attention	SDMT (SD)	41.0(10.7)	40.0(10.9)	0.469
	TMT-A, s (SD)	43.25(10.43)	45.40(13.81)	0.157
Executive-function	TMT-B, s (SD)	77.35(13.64)	83.14(17.91)	0.004*
	VST interference, s (SD)	13.14(1.73)	13.81(1.87)	0.003*
Visual space	CDT (SD)	11.1(1.6)	10.6(2.0)	0.032*
Affective-disorder	HAMA (SD)	2.1(1.5)	2.6(1.9)	0.039*
	HAMD (SD)	3.6(2.0)	3.2(1.9)	0.180
Serum $pTau_{181}$，pg/ml (SD)		3.11(0.35)	3.21(0.34)	0.022*
Serum $A\beta_{40}$，pg/ml (SD)		103.60(28.59)	110.79(28.72)	0.043*
Serum $A\beta_{42}$，pg/ml (SD)		74.98(18.78)	81.49(23.21)	0.013*
Serum total Aβ，pg/ml (SD)		178.58(29.93)	192.28(51.02)	0.009*
Serum $A\beta_{42/40}$ ratio (SD)		0.79(0.33)	0.73(0.78)	0.045*
Serum SOD，U/mL (SD)		46.05(3.32)	44.88(3.24)	0.004*
Serum MDA，nmol/mL (SD)		13.80(1.60)	14.24(1.50)	0.023*
Serum GR，U/L (SD)		40.46(1.69)	40.19(1.69)	0.192
Serum GPx，μmol/L (SD)		663.25(44.51)	649.53(42.81)	0.011*

MoCA, Montreal Cognitive Assessment; HVLT, Hopkins Verbal Learning Test; SDMT, Symbol Digit Modalities Test; TMT, Trail Making Test; VST, Victoria Stroop Test; CDT, Clock Drawing Test; HAMA, Hamilton Anxiety Rating Scale; HAMD, Hamilton Depression Rating Scale; $pTau_{181}$, phosphorylated tau-181; Aβ, Amyloid-β; SOD, superoxide dismutase; MDA, Malondialdehyde; GR, Glutathione reductase; GPx, Glutathione peroxidase. *$P<0.05$ was defined as being statistically significant.

2 Data Collection Questionnaire

Participant Number:_____________ Inclusion Time:______Month______Day______Year

General information

Name:_________ Age:__________ Gender:__________ Occupation:__________.
Education Level (Elementary school / Junior high school / High school / University and Above)
Telephone number:_______________ Address:___________________________________.
Height:_______m Weight:______kg BMI (kg/m^2):______ Blood Pressure:______mmHg
History of Hypertension: (Yes No) Duration:_____years
History of Diabetes: (Yes No) Duration:_____years
History of Hypercholesterolemia: (Yes No) Duration:_____years
History of Atrial Fibrillation: (Yes No) Duration:_____years
Smoking History: (Ever / Now / No) Duration:_____years
Alcohol Consumption: (Ever / Now / No) Duration:_____years
Any Psychotropic Drugs Used in the Past Year: (Yes No)
Physical Activity Scores (Sum of the Six Activities):_____scores

	2 scores		1 score	0 score	
	Each Day	Several Times per Week	Once a Week	Less than Once a Week	Never
Bicycling					
Walking					
Swimming					
Gymnastics					
Chores/Gardening					
Others					

Tea consumption information

The habit of tea consumption	Yes √ No ×			
The type of tea most commonly consumed	Green tea	Black tea	Oolong tea	Others
The consumption frequency	Days/week			
The daily volume of tea	Cups/day<br>(A cup of standard green tea has a volume of 100ml)			
The duration of tea consumption habit	Years			

Measurements of Serum Biomarkers Levels

Amyloid-β_{40}:______pg/ml Aβ_{42}:______pg/ml A$\beta_{40/42}$:______ Total Aβ:______pg/ml
Phosphorylated Tau-181:______pg/ml
Superoxide Dismutase (SOD): ______U/mL
Malondialdehyde (MDA): ______nmol/mL
Glutathione Reductase (GR): ______U/L
Glutathione Peroxidase (GPx): ______μmol/L
Fasting Glucose: ______mmol/L
Cholesterol: ______mmol/L
Triglyceride: ______mmol/L
Homocysteine: ______umol/L

Cerebral MRI or CT: ____.

Assessment of Cognitive Function

**Cognitive Domain**	**Scale**	**Result**
Global function	Montreal Cognitive Assessment (MoCA)	Score: /30
Memory	Hopkins Verbal Learning Test-Revised (HVLT-R)	Immediate Recall: /36
		Delayed Recall: /12
		Delayed Recognition: /24
Language	Verbal Fluency Test	Score:
Attention	Symbol Digit Modalities Test	Score:
	Trail Making Test-A	Score: Time:
Executive Function	Victoria Stroop Test (VST)	Score: Time:
		Score: Time:
		Score: Time:
	Trail Making Test-B	Score: Time:
Visual Space	Clock Drawing Test	Score: /15
Affective Disorder	Hamilton Anxiety Rating Scale	Score:
	Hamilton Depression Rating Scale	Score:
Functional Autonomy	Instrumental Activities of Daily Living (IADL)	Score:
